# Structural Characterization of Cuta- and Tusavirus: Insight into Protoparvoviruses Capsid Morphology

**DOI:** 10.3390/v12060653

**Published:** 2020-06-17

**Authors:** Mario Mietzsch, Robert McKenna, Elina Väisänen, Jennifer C. Yu, Maria Ilyas, Joshua A. Hull, Justin Kurian, J. Kennon Smith, Paul Chipman, Yi Lasanajak, David Smith, Maria Söderlund-Venermo, Mavis Agbandje-McKenna

**Affiliations:** 1 Department of Biochemistry and Molecular Biology, Center for Structural Biology, McKnight Brain Institute, College of Medicine, University of Florida, Gainesville, FL 32610, USA; mario.mietzsch@ufl.edu (M.M.); rmckenna@ufl.edu (R.M.); jennifer.yu@ufl.edu (J.C.Y.); mariailyas@ufl.edu (M.I.); the.hegemon@ufl.edu (J.A.H.); jkurian@udel.edu (J.K.); k.smith@stridebio.com (J.K.S.); pchipman@ufl.edu (P.C.); 2 Department of Virology, University of Helsinki, 00014 Helsinki, Finland; elina.vaisanen@helsinki.fi (E.V.); maria.soderlund-venermo@helsinki.fi (M.S.-V.); 3 Emory Comprehensive Glycomics Core, Emory University School of Medicine, Atlanta, GA 30322, USA; ylasana@emory.edu (Y.L.); dfsmith@emory.edu (D.S.)

**Keywords:** parvovirus, protoparvovirus, cryo-EM, capsid, human pathogen, glycan receptor, sialic acid

## Abstract

Several members of the *Protoparvovirus* genus, capable of infecting humans, have been recently discovered, including cutavirus (CuV) and tusavirus (TuV). To begin the characterization of these viruses, we have used cryo-electron microscopy and image reconstruction to determine their capsid structures to ~2.9 Å resolution, and glycan array and cell-based assays to identify glycans utilized for cellular entry. Structural comparisons show that the CuV and TuV capsids share common features with other parvoviruses, including an eight-stranded anti-parallel β-barrel, depressions at the icosahedral 2-fold and surrounding the 5-fold axes, and a channel at the 5-fold axes. However, the viruses exhibit significant topological differences in their viral protein surface loops. These result in three separated 3-fold protrusions, similar to the bufaviruses also infecting humans, suggesting a host-driven structure evolution. The surface loops contain residues involved in receptor binding, cellular trafficking, and antigenic reactivity in other parvoviruses. In addition, terminal sialic acid was identified as the glycan potentially utilized by both CuV and TuV for cellular entry, with TuV showing additional recognition of poly-sialic acid and sialylated Lewis X (sLeXLeXLeX) motifs reported to be upregulated in neurotropic and cancer cells, respectively. These structures provide a platform for annotating the cellular interactions of these human pathogens.

## 1. Introduction

The members of the *Parvoviridae* are linear, non-segmented, single-stranded DNA viruses, with a genome of ~4–6 kb [1]. Parvoviruses are among the smallest viruses (hence the name, from the Latin word parvus meaning small) with a non-enveloped capsid of 215–260 Å in diameter. They infect a wide range of hosts, reflected by the three subfamilies: the members of the *Parvovirinae* infect vertebrates, those of the *Densovirinae* infect arthropods, and those of the *Hamaparvovirinae* infect either vertebrates or invertebrates [1,2]. The *Parvovirinae* subfamily is further divided into ten genera; *Amdoparvovirus*, *Artiparvovirus*, *Aveparvovirus*, *Bocaparvovirus*, *Copiparvovirus*, *Dependoparvovirus*, *Erythroparvovirus*, *Loriparvovirus*, *Protoparvovirus*, and *Tetraparvovirus* [1]. To date, five of the ten genera in this subfamily contain viruses capable of infecting humans: *Bocaparvovirus* (e.g., human bocavirus 1 [HBoV1]), *Dependoparvovirus* (e.g., adeno-associated virus 2 [AAV2]), *Erythroparvovirus* (e.g., parvovirus B19), *Protoparvovirus* (e.g., bufavirus 1 [BuV1]), and *Tetraparvovirus* (e.g., human parvovirus 4). Currently, no known human-infecting virus has been identified in the *Amdoparvovirus*, *Artiparvovirus*, *Aveparvovirus*, *Copiparvovirus,* or *Loriparvovirus* genera. The most recently identified emerging human pathogens of the *Parvoviridae* family belong to the *Protoparvovirus* [3,4,5].

Protoparvoviruses, have two major open reading frames (ORFs) under the control of the p6 and p38 promotors, which encoded the non-structural (NS) and capsid viral proteins (VPs), respectively [6]. The VP ORF encodes two overlapping structural proteins, VP1 and VP2, sixty copies of which assemble the T = 1 icosahedral capsid in an approximate 1:10 ratio, respectively. The VP1, ~80 kDa, is the larger but minor capsid component containing a unique N-terminal extension termed VP1u compared to VP2, ~64 kDa, which is the major capsid component [6]. For some members of this genus, a third VP, VP3, is produced by proteolytic cleavage of the N-terminal 15–18 amino acids of VP2 following genome packaging [7]. This then becomes the major capsid VP. In the icosahedral capsid the VPs assemble utilizing 2-, 3-, and 5-fold symmetry-related VP interactions [8]. The individual VPs contain an eight-stranded (βB to βI) anti-parallel β-barrel motif, with the βBIDG sheet forming the interior surface of the capsid. In addition, a βA strand that runs anti-parallel to the βB strand and a conserved helix, αA, located between strands βC and βD, are also part of the conserved vertebrate parvovirus core structure [6]. Large loops inserted between the β-strands form the surface topology of the capsid. These loops are named after the β-strands that they connect, for example, the HI loop connects the βH and βI strands. While the VP core structure is conserved, these surface loops display structural variability between parvoviruses of different genera and within the same genus, especially at their apex, termed variable regions (VRs). For the protoparvoviruses ten VRs have been described, VR0 to VR8 including VR4a and VR4b, which are defined as regions with two or more amino acids with Cα positions greater than 2 Å apart when the VPs of different protoparvoviruses are superposed. The VP amino acid sequence varies significantly between the protoparvoviruses with sequence identities ranging from 29–95% [9]. The type member of this genus is the prototype strain of minute virus of mice (MVMp), a rodent pathogen [10].

Recent advances in DNA sequencing technology have led to the discovery of three new members of the *Protoparvovirus* genus capable of infecting humans; namely bufavirus, (BuV), cutavirus (CuV), and tusavirus (TuV) [3,4,5,11]. Of these protoparvovirus, BuV1 and 2 were first discovered in 2012 in the feces of children from Burkina Faso suffering from diarrhea and a child with non-polio acute flaccid paralysis from Tunisia [4], and later in diarrheal samples from adults in Finland and Holland [12,13]. Similarly, in 2014, TuV was detected in feces of a Tunisian child with unexplained diarrhea [3] and TuV IgG in one child and one adult from Finland [14,15]. In 2016, CuV was found in human fecal samples and cutaneous T-cell lymphomas (CTCL) [5,11]. While CuV DNA was found in skin biopsies from ~5% of German and 16% of Finnish patients with CTCL [16,17], it was absent in healthy patients, providing a statistically significant association to CTCL [17]. It has been infrequently detected also in malignant skin melanomas and carcinomas [11,17,18]. Recently, a study for BuV, CuV, and TuV found low prevalences of BuV IgGs in adults in Finland (~2%) and the United States (~4%), but high prevalences in Iraq (~85%), Iran (~56%), and Kenya (~72%), whereas the CuV IgG prevalences were low (0%–6%), and TuV IgGs undetected in the cohorts tested [14].

The virus capsid plays a central role in the infection by the protoparvoviruses as it mediates the attachment of the virus to specific receptors on target cells. For the protoparvoviruses, sialic acid (SIA) was reported as the infectious receptor for MVM, H1-PV, and PPV, and a hemagglutination receptor for CPV and FPV and with the binding site mapped near the 2-fold symmetry axis of their capsids [19,20,21,22]. The capsid also protects the genome enroute to the nucleus for uncoating and replication. This study determines the capsid structures of TuV and CuV using cryo-electron microscopy and image reconstruction (cryo-EM) at ~2.9 Å resolution and compares them to the recently determined structure of BuV and other protoparvoviruses toward functional annotation. The high resolution of the maps enabled the atomic assignment of the amino acids of the major capsid protein, VP2. These viruses, which display low sequence identities, share common capsid features with other parvoviruses. Furthermore, the overall VP2 structure topologies are similar, with the core eight-stranded β-barrel superposable. However, major differences are localized within the surface loops, in previously defined VRs. These have been shown to be involved in receptor binding, cellular trafficking, transcription, and antigenic reactivity. In addition, terminal SIA was identified as the potential attachment glycan receptor for both CuV and TuV. These studies provide a structural platform for functional annotation of these human pathogens that will help to understand their disease mechanisms on a molecular level. This information could be applicable toward their use as gene delivery tools or the development of therapeutics.

## 2. Materials and Methods

### 2.1. Production and Purification of CuV and TuV Virus-Like Particles

The CuV and TuV VP2 genes were cloned into the pFastBac1 plasmid to generate recombinant baculoviruses that express virus-like particles (VLPs) using the Bac-to-Bac system according to the manufacturer’s instructions (Invitrogen, Carlsbad, CA, USA) [23]. *Sf9* insect cells, maintained in SFM Sf9-900 II medium (ThermoFisher, Waltham, MA, USA) supplemented with 1% antibiotic-antimycotic (ThermoFisher) at 28 °C, were infected with the recombinant baculoviruses at a multiplicity of infectivity (MOI) of 5 and harvested 72 h post infection by centrifugation at 2000 rpm for 20 min at 4 °C. The pellets were re-suspended in lysis buffer (25 mM Tris-HCl pH 8.0, 100 mM NaCl, 0.2% Triton X-100, 2 mM MgCl_2_) (TNTM buffer) and frozen and stored at −20 °C until purification. For purification, VLPs were released from the infected cell pellet by three freeze-thaw cycles. Following the freeze-thaws, benzonase (Millipore, Burlington, MA, USA) was added and the sample incubated for 30 min at 37 °C. One microliter of benzonase per 10 mL of pellet supernatant (activity of 1 × 10^6^ U/mg of protein) was used for this reaction. Cellular debris was pelleted by centrifugation at 10,000 rpm (Beckman JA-20) for 15 min at 4 °C. The resulting clarified supernatants were subjected to a 20% (*w*/*v*) sucrose cushion in 50 mM Tris-HCl pH 8.0, 100 mM NaCl, 1 mM EDTA, and 0.2% Triton X-100 (TNET buffer) to pellet the VLPs by ultracentrifugation at 45,000 rpm (Beckman 70-Ti) for 3 h at 4 °C. The resulting pellets were resuspended overnight in TNTM. The samples were further purified using sucrose gradients (5 to 40% sucrose in TNTM) ultracentrifugation at 35,000 rpm (Beckman SW40-Ti) for 3 h at 4 °C. Visible bands were extracted at 20% for both viruses, and dialyzed against 1× phosphate buffer saline (PBS) (2.8 mM KCl, 137 mM NaCl, 10 mM Na_2_HPO_4_, 1.8 mM KH_2_PO_4_) at 4 °C. Concentrations (in mg/mL) were determined based on UV absorbance of 280 nm with an extinction coefficient of 1.7 M^−1^ cm^−1^. The purified virus samples were concentrated to ~1 mg/mL using Apollo concentrators (Orbital Biosciences, Topsfield, MA, USA) for further characterization and structure determination.

### 2.2. VLP Sample Purity and Integrity

The purity and integrity of the VLPs were confirmed by sodium dodecyl sulfate polyacrylamide gel electrophoresis (SDS-PAGE) and negative-stain electron microscopy (EM), respectively. For the SDS-PAGE analysis, the samples were incubated with 1× Laemmli sample buffer (Bio-Rad, Hercules, CA, USA) with 2% β-mercaptoethanol and boiled for 5 min at 100 °C. The denatured VLPs were applied to a 10% polyacrylamide gel and ran at 80 V. The gel was washed three times with distilled water (diH_2_O) and stained with GelCode blue protein stain (Invitrogen) for 30 min. The gel was de-stained with diH_2_O prior to imaging using a GelDoc XR+ system (Bio-Rad). For negative stain EM, carbon-coated copper EM grids (Ted Pella, Redding, CA, USA) were incubated with 5 µl of 1:10 diluted sample for 1–5 min, washed with diH_2_O, and stained with 2% uranyl acetate for 6 s. The grids were imaged on a Tecnai G2 Spirit TEM (FEI Co, Hillsboro, OR, USA) microscope operated at an accelerating voltage of 120 kV and micrographs were collected on a Gatan 2K × 2K CCD camera.

### 2.3. Cryo-Electron Microscopy (Cryo-EM) and Image Reconstruction

Three microliters of purified CuV and TuV VLPs (~1 mg/mL) were applied to C-flat holey carbon grids (Protochips, Inc., Morrisville, NC, USA) and vitrified using a Vitrobot™ Mark IV (FEI Co). The capsid distribution and ice quality of the grids were examined using a 16-megapixel CCD camera (Gatan, Inc., Pleasanton, CA, USA) in a Tecnai (FEI Co.) G2 F20-TWIN transmission electron microscope operated at a voltage of 200 kV using low dose conditions (~20 e/Å^2^). Optimal grids were used for collecting micrograph movie frames using the Leginon semi-automated application on a Titan Krios electron microscope (FEI Co.) operated at 300 kV with images recorded on a Gatan K2 Summit direct electron detection camera for CuV and TuV. The microscope was equipped with a Gatan post-column imaging filter (GIF) utilizing a slit width of 20 eV. Data collection used counting mode and an accumulated dose of 75 e−/Å^2^ fractionated into 50 movie frames per micrograph. Movie frame alignment used the MotionCor2 application with dose weighting [24]. The data sets were collected as part of the National Institutes of Health (NIH) “West/Midwest Consortium for High-Resolution Cryo Electron Microscopy” project. A nominal magnification of 130,000× was used for data collection resulting in a pixel size of ~1.1 Å. The data collection parameters are provided in Table 1. For the three dimensional image reconstruction of the CuV and TuV data the cisTEM software package was utilized [25]. Briefly, the aligned micrographs were imported into the program and their contrast transfer function (CTF) parameters estimated. The CTF information was used to eliminate micrographs of poor quality. This was followed by automatic capsid picking using a radius of 125 Å. This set of capsids was subjected to 2D classification that eliminated ice particles and debris from the automatic picking process. Following 2D classification, the structures of the CuV and TuV capsids were reconstructed using default settings. This included ab initio 3D model generation, auto refinement, and density map sharpening with a pre-cut off (low resolution amplitudes) B-factor value of -90 Å^2^, and variable post-cut off (high resolution amplitudes) B-factor values such as 0, 20, and 50 Å^2^. The sharpened density maps were inspected in the Coot and Chimera applications [26,27]. The -90 Å^2^ (pre-cut off) sharpened maps were used for assignment of the amino acid main- and side chains. The resolution of the cryo-reconstructed maps for CuV and TuV were estimated to be 2.87 and 2.88 Å, respectively, based on a Fourier shell correlation (FSC) of 0.143 (Table 1). 

Initial CuV and TuV VP2 atomic models were generated using the deposited capsid structure of BuV2 (PDB ID: 6BX0) as a template in Swiss Model [28], with VP residues changed to their respective viruses. The VLP 60mer generated using the ViperDB server [29] were oriented and positioned into the Cryo-EM maps of CuV and TuV using the “Fit in Map” option in Chimera while maximizing the correlation coefficient (CC). The EMAN2 subroutine e2proc3d.py was implemented to resize the maps based on best-fit parameters as determined by CC from Chimera [27,30]. The 60mer models and maps were visualized using Coot and the positions and conformations of Cα-backbone and side-chains of residues were manually adjusted and real-space refined [26]. In an alternating manner, the modified CuV and TuV VP2 capsids were further refined against the cryo-EM maps using Coot and PHENIX until convergence [31]. Visual representations of maps and models were generated using UCSF Chimera [27]. Final statistics for the CuV and TuV VP2 coordinates are provided in Table 1. 

### 2.4. Sequence and Structural Comparison

The surface morphology of the CuV, MVMp and TuV capsids were visually compared using Chimera [27] while the VP2 models were superposed in Coot to obtain overall paired RMSDs between Cα positions and to identify regions of structural similarities and differences. Deviations between non-overlapping Cα positions, because of residue deletion/insertions, were measured using the distance tool in Coot. Regions of two or more adjacent amino acids with ≥2.0 Å difference in superposed VP2 Cα position were considered to be structurally diverse and assigned to a VR. This information was also used for a structure-based sequence alignment of CuV and TuV compared to the MVMp VP structure, and to calculate the structural identity (in %) that was defined as the number of aligned residues (≤2.0 Å apart) divided by the total number of residues. Amino acid sequence alignments of the different protoparvoviruses were done utilizing the sequence alignment option in VectorNTI (Invitrogen).

### 2.5. Fluorescent Labeling of VLPs

CuV and TuV VLPs were fluorescently labeled using the DyLight 488 antibody labeling kit (Thermo Fisher) following a modified version of the manufacturer’s protocol. A total of 40 μL of borate buffer (0.67 M, pH 8.5) was added to 500 μL of the VLPs at a concentration >0.5 mg/mL, mixed and transferred to the DyLight reagent vial. The mixture was incubated for 1 h at RT protected from light. Unbound fluorescent molecules were removed from the sample by dialysis using a membrane with a 30 kDa cutoff into 4 L of 1× PBS. The dialysis was performed at 4 °C with slow stirring utilizing a magnetic stirrer. The dialysis buffer was changed two additional times after 3 h of dialysis. The success of the labeling procedure was confirmed by SDS-PAGE showing fluorescent VP2 bands when viewed under UV light.

### 2.6. Cell Binding Assay

Low passage Chinese hamster ovary (CHO) cell lines Pro5 and Lec2 were cultured as monolayers in MEM-α (ATCC) with 10% FBS (fetal bovine serum) and 1% antibiotic-antimycotic in a 5% CO_2_ 37 °C incubator. For cell binding assays, the CHO cells were detached from plates by addition of EDTA, pelleted, resuspended in MEM-α to 5 × 10^5^ cells/mL, pre-chilled for 30 min at 4 °C, and aliquoted to 500 µL fractions. Each tube of cells was then incubated with the fluorescently labeled VLPs at a MOI of 10^6^ under constant rotation for 3–4 h at 4 °C (protected from light). Following the incubation, the cells were pelleted at 2000 rpm (Beckman JA-10) for 10 min and the supernatant discarded. Unbound VLPs were removed by washing the cells with 300 µL ice-cold 1× PBS, followed by centrifugation. Pellets were resuspended in 300 µL 1× PBS and analyzed utilizing a FACS Canto (BD, Franklin Lakes, NJ, USA). Cells without added fluorescent-labeled capsids were used as baseline to determine the percentage of fluorescent cells for the other samples. All experiments were conducted in triplicate. The FSC Express5 software suite (De Novo Software, Pasadena, CA, USA) was used to analyze the raw data.

### 2.7. Glycan Array Screening

Fluorophore-labeled VLPs were analyzed on glycan microarrays for their glycan-binding ability at the Emory Comprehensive Glycomics Core. The procedure was described previously [32]. In brief: 600 different glycan structures are printed on microscope glass slides (CFG glycan array V5.2), each in replicates of six. The samples at ~180 µg/mL, supplemented with 1% BSA and 0.05% Tween-20, were incubated on the glycan array for 1 h at room temperature in a dark humidified chamber. The slides were washed in PBS with 0.05% Tween-20, dried by spinning, and scanned by an Innopsys scanner using the 488 nm wavelength laser. The data sets were analyzed by averaging the data for four replicates after elimination of two spots with the highest and lowest intensity.

### 2.8. Protein Data Bank Accession Numbers

The cryo-EM reconstructed density maps and model coordinates for CuV and TuV were deposited into the EMDB database with the accession numbers EMD-22008 and EMD-22010, and PDB-IDs 6X2I and 6X2K, respectively.

## 3. Results and Discussion

### 3.1. CuV and TuV VLPs Were Generated at Levels Suitable for Structure Determination

**Figure 1:**
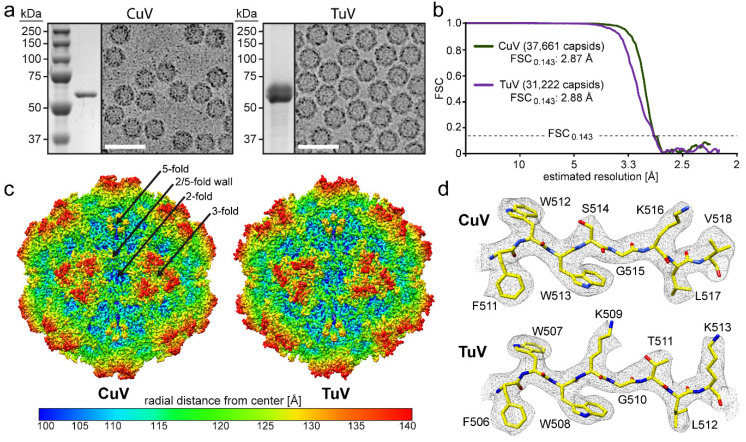
CuV and TuV samples and structures. **(a)** SDS-PAGE of purified CuV and TuV with a band at ~60 kDa equivalent to the size of VP2 and example cryo-electron micrograph. Scale bar: 50 nm. **(b)** Fourier shell correlation (FSC) plotted against resolution for the reconstructed CuV and TuV structures. The resolution of the maps were estimated to be 2.87 and 2.88 Å resolution, respectively, based on an FSC threshold of 0.143. **(c)** The capsid surface density maps contoured at a sigma (σ) threshold level of 1.5. The maps are radially colored (blue to red) according to distance to the capsid center, as indicated by the scale bar below. The approximate icosahedral 2-, 3-, and 5-fold axes are indicated on the CuV capsid map. **(d)** CuV and TuV residues modelled for the βI strand are shown inside their density maps (in grey). The amino acid residues are as labeled and shown as stick representation and colored according to atom type: C = yellow, O = red, N = blue. Panel (c) and (d) were generated using UCSF-Chimera [27].

Recombinant baculoviruses carrying the CuV and TuV *cap* genes expressing VP2 were used for the production of CuV and TuV VLPs in *Sf*9 cells. Following sucrose cushion and gradient ultracentrifugation, the CuV and TuV preparations were analyzed for their purity by SDS-PAGE which revealed single bands at ~60 kDa corresponding to the size of VP2 (Figure 1a). Cryo-EM micrographs showed assembled capsids of approximately 25 nm in diameter without the presence of contaminating proteins (Figure 1a). Thus, the samples were deemed suitable for atomic resolution structure determination and movie frame micrographs were collected at the West/Midwest Consortium for high-resolution cryo electron microscopy. 3D image reconstruction of the data utilizing 15,296 and 33,191 capsids resulted in structures with an estimated resolution of 2.87 and 2.88 Å based on an FSC threshold of 0.143 for CuV and TuV, respectively (Figure 1b, Table 1). The reconstructed CuV and TuV maps displayed familiar surface features of other *Parvovirinae* subfamily members such as channels at the icosahedral 5-fold axes, protrusions surrounding the 3-fold axes, and depressions at the 2-fold axes and surrounding the 5-fold axes. The depressions surrounding the 2- and 5-fold axes are separated by a raised region termed the 2/5-fold wall (Figure 1c).

The high-resolution density maps for CuV and TuV allowed unbiased modeling of the atomic VP2 structure (Figure 1d). The first ordered amino acid at the N-terminus of VP2 was glycine 32 in the case of CuV and alanine 19 for TuV (VP2 numbering). Despite the different residue number and type, they are located at the same position within the VP monomers. The lack of ordering of the remainder of the N-terminus is consistent with reports for all other current capsid structures within the *Parvovirinae* regardless of whether cryo-EM or X-ray crystallography was utilized for determination or whether VP1u was present, likely because of the presence of a glycine-rich domain at the N-terminus [33]. This disorder has been proposed to confer flexibility to the VP1u region allowing its externalization through the 5-fold channel during the viral replication cycle [34]. Following the first ordered residue, the main and side chains are well defined (Figure 1d) to the C-terminus for both viruses. However, one exception was the diffuse electron density observed for amino acids 533–534 exclusively in the TuV map located near the 2-fold depression where only weak density was present for the main chain at a sigma (σ) threshold of one preventing reliable placement of amino acid side chains. The less ordered state in this region of the TuV map could be the result of the presence of multiple smaller amino acids 531-GAAV-534 conferring flexibility to this loop. The final VP models, refined in the context of their respective 60mers relative to the cryo-reconstructed maps, had high CC and good geometry values comparable to other virus structures determined to similar resolution (Table 1) [9,33,35,36,37].

### 3.2. Comparison of CuV and TuV to other Protoparvoviruses Suggest Host-Driven Capsid Surface Morphologies

**Figure 2:**
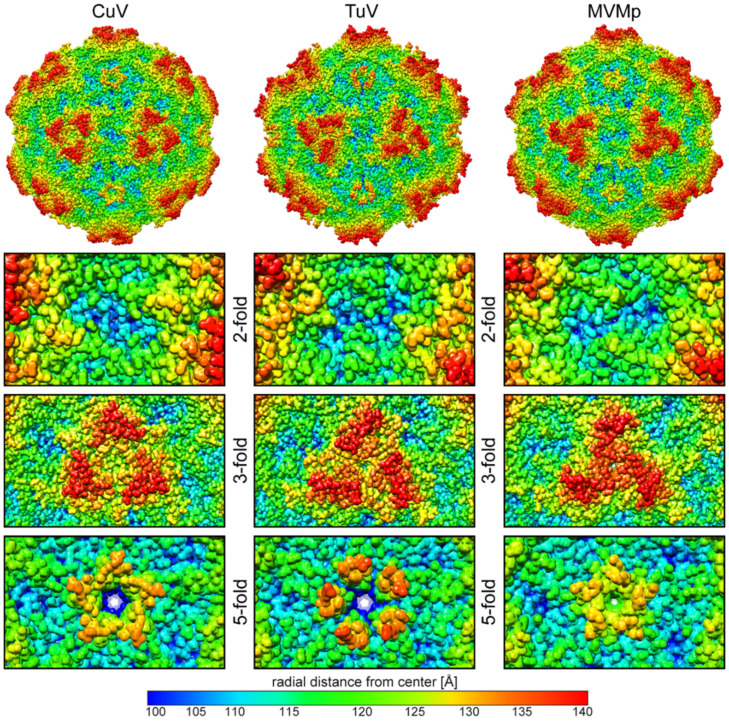
The CuV, TuV, and MVMp capsid surface. Surface representation of the atomic models built into the density maps of CuV, TuV, and MVMp [41] (PDB-ID: 1Z14) and radially colored (blue to red) according to distance from the capsid center, as indicated by the scale bar below. The entire capsid and close-up views of the icosahedral 2-, 3-, and 5-fold symmetry axes are shown. This figure was generated using UCSF-Chimera [27].

Several capsid structures are available for the protoparvoviruses, including for MVMp, the type member of this genus [9,37,38,39,40,41,42]. The amino acid sequence identity of the ordered VP region of CuV and TuV compared to MVM is low at 31.8 and 41.4%, respectively. Of note, the sequence identity between CuV and TuV is also low at 33.3%, implying that the three viruses are equally different from each other. Nonetheless, the core features of the capsids are conserved with respect to other parvoviruses as mentioned above. Instead, on the capsid surface structural differences are observed between the viruses at the depressions, protrusions, and channels (Figure 2). In the CuV capsid, the depression at the 2-fold (blue to cyan colors, corresponding to a distance of <110 Å from the center of the capsid) is broad, continuous, and has a slight diagonal tilt relative to the icosahedral 2-fold axes. In contrast, the depression in the MVMp and TuV capsids are two and four smaller depressions, respectively, on either side of the 2-fold axes, with the outer TuV depressions extended toward the 2/5-fold wall in TuV. The protrusions (orange to red colors, corresponding to a distance of >130 Å from the center of the capsid) around the 3-fold symmetry axis display the most striking differences for these capsids. While clearly separated in the CuV capsid and completely merged into a single “spinwheel” in MVMp, the 3-fold protrusions in the TuV capsid displayed an intermediate phenotype, with the protrusion almost fused at the 3-fold axis (Figure 2). TuV exhibits the most extended 5-fold channel among the known capsid structures of all members of the Parvovirinae with a radial distance of ~135 Å from the center of the capsid. The DE loop assembling the 5-fold channel in the three viruses compared adopt different conformations at the apex. In CuV and MVMp, the apex of the loop is splayed outwards resulting in a radial distance of ~132 and ~130 Å, respectively (Figure 2). In addition, the pore of the 5-fold channel is narrower in MVMp compared to CuV and TuV, especially at a radial distance of ~120 Å from the center of the capsid. 

### 3.3. The CuV and TuV Capsids Exhibit the Most Differences in the Variable Regions of the Protoparvoviruses

**Figure 3:**
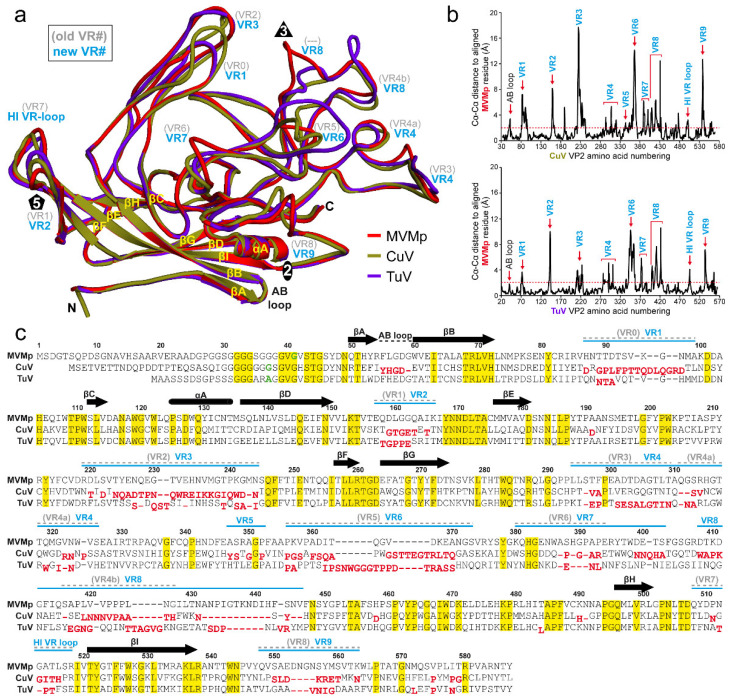
Structural comparison of the CuV, TuV, and MVMp VPs. (**a**) Structural superposition of MVMp (red), CuV (olive), and TuV (purpleblue) shown as ribbon diagrams. The positions of β-strands A-I, α-A, the N- and C-terminus, and approximate icosahedral 2-, 3-, and 5-fold axis are indicated. For the VRs, the previous nomenclature (in gray and in parenthesis) and the new assignment in blue is given. This figure was generated using PyMol [45]. (**b**) Cα-Cα distance plot for the CuV (top) and TuV (below) residues relative to MVMp when the VP structures are superposed. Regions with high structural variability are labeled with the new VR (blue) assignment. (**c**) Structure-based sequence alignment of CuV and TuV compared to MVMp, except for the VP2 N-termini prior to the first structurally ordered amino acid (green). The VP2 residue numbers indicated above the amino acid sequence are based on MVMp. Secondary structure elements such as β-strands and α-helices, are shown as black arrows and black cylinders, respectively. The positions of the VRs are indicated above the sequence as in (**a**). Amino acids highlighted in yellow indicate sequence identity among all three viruses. Amino acids whose Cα atoms are further than 2 Å apart when superposed onto MVMp are shown offset and in red below the aligned residues.

To annotate observed capsid surface differences among the protoparvoviruses (Figure 2), and potentially functional regions, the VP models of CuV and TuV were superposed onto that of MVMp (PDB-ID: 1Z14), whose capsid was functionally annotated previously [21,43,44]. This superposition resulted in an overall RMSD of 3.0 Å for CuV vs. MVMp, 2.2 Å for TuV vs. MVMp, and 2.7 Å for CuV vs. TuV (Table 2). These numbers are substantially higher than for previously reported protoparvovirus VP structure comparisons, with maximum RMSDs of ~1.0 Å [37,39]. Despite the higher RMSD values, CuV and TuV conserve the core β-strands (βA–βI) and α-helix A (αA) which are superposable to MVMp (Figure 3a). As such, the core of the VP structure (without VRs) shows comparative low RMSD values between 0.8 and 1.0 Å (Table 2). High structural variability was observed in the surface loops (Table 2, Figure 3a and b). In some VP regions, the Cα-Cα distance between aligned structures exceeded 17 Å in the case of CuV vs. MVMp, and up to 11 Å for TuV vs. MVMp (Figure 3b). While most of the observed structural differences were located within previously defined VRs [39], some of the structural variability was detected outside of these previously defined regions, such as in the AB-loop, between VR6 and VR4b, and between VR4b and βH (Figure 3c, gray VR numbering). 

**Figure 4:**
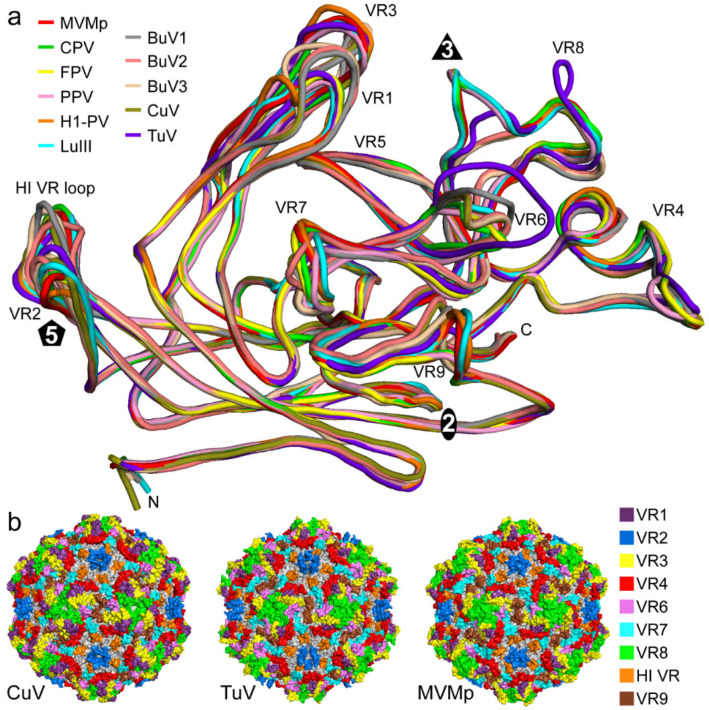
Structural comparison of protoparvoviruses. (**a**) The superposed VP structures for all available members are shown as coil diagrams: MVMp (red), CPV (green), FPV (yellow), PPV (pink), H1-PV (orange), LuIII (cyan), BuV1 (gray), BuV2 (salmon), BuV3 (tan), CuV (olive), and TuV (purpleblue). The N- and C-terminus, the approximate icosahedral 2-, 3-, and 5-fold axis, and the VRs are indicated. (**b**) Surface representations of the CuV, TuV, and MVMp capsids. The new VR assignment is used and colored as indicated in the legend on the right. This figure was generated using PyMol [45].

**Figure 5:**
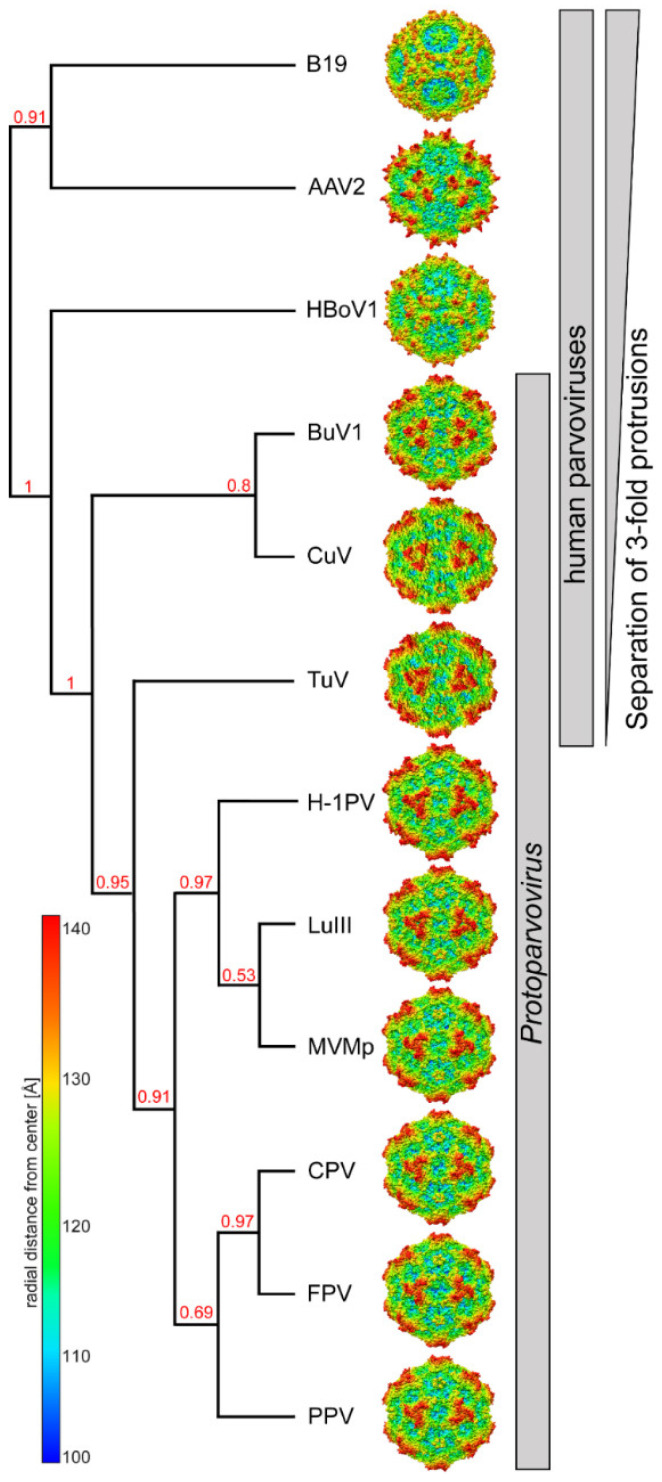
Cladogram showing the capsid amino acid and structural relationship between the protoparvoviruses and selected members of human parvoviruses. B19: parvovirus B19; AAV: adeno-associated virus; HBoV: human bocavirus. This image was generated online (http://www.phylogeny.fr/) utilizing the VP2/3 sequences as input. Branch support values are given. Radially-colored capsid surface representations (blue to red, as shown in the scale bar on the left) are viewed along the 2-fold axis and generated using Chimera [27].

Because of these new “variabilities” compared to the structurally conserved regions we propose a new assignment of the protoparvovirus VRs (Figure 3, blue VR numbering) following numerical order that is also more comparable to the VRs of the dependo- and bocaparvoviruses [6]. The 5-fold region consists of VR2 (previously VR1) and the HI VR loop (previously VR7). The HI VR loop surrounds the 5-fold channel and forms the floor of the depression at the base. CuV is structurally more similar to MVMp in this loop with a local RMSD of 2.0 Å compared to TuV at 2.3 Å (Table 2). This is likely due to a deletion in TuV relative to CuV and MVMp (Figure 3c). The five VR2 are at the top of the DE loops forming the five-fold channel. The extended channel in TuV (Figure 2) is not the result of amino acid insertions in the loop, but is instead caused by a different structural configuration of the DE loops which runs more radially upright from the capsid surface, compared to MVMp where the apex of the loop is tilted (Figure 3a). The narrow channel in MVMp is caused by Q158 pointing toward the 5-fold axis whereas CuV and TuV both have a smaller threonine side chain in this position. Leucine 172, which is located in the interior of the five-fold channel was previously described to be important for DNA packaging and for VP1u externalization for MVM [46]. This residue is conserved in both CuV and TuV. In addition, MVMp’s lysine 153, which resulted in an assembly defect when mutated to alanine [47], is conserved in CuV and TuV. The 3-fold region is composed of VR1 (previously VR0), VR3 (previously VR2), and VR8 (previously VR4b). In CuV, all three VRs have significant structural differences compared to MVMp, with local RMSDs between 4.1 to 6.6 Å (Figure 3b, Table 2). While CuV’s VR1 is extended compared to MVMp due to a five amino acid insertion, VR3 is one amino acid shorter and adopts a different conformation (Figure 3a,c). In TuV, both VR1 and VR3 display less variability to MVMp (local RMSD 1.6 – 2.2 Å) with no insertion or deletion in VR1 and a single amino acid deletion in VR3 (Figure 3a,c). In contrast, VR8 shows a substantial structural difference in both CuV and TuV compared to MVMp (Figure 3a). Previously, only a short region of this loop was defined as a VR (VR4b) but CuV has two additional amino acid regions flanking the former VR4b that are independent VRs (Figure 3a,c). These three regions have now been combined and renamed VR8. The C-terminal flanking region of the former VR4b, a loop inserted in MVMp (11 amino acids) that is orientated toward the 3-fold axis, is absent in CuV (Figure 3a,c). This region is responsible for the striking different surface appearance of the 3-fold region (Figure 2). The absence of this loop separates the 3-fold protrusions in the CuV capsid while the loop merges the 3-fold protrusions in MVMp as well as protoparvoviruses CPV, FPV, PPV, H1-PV, and LuIII, which all share this loop (Figure 2, Figure 4 and Figure 5) [37,38,39,40,41,42]. Similar to CuV, the BuVs do not have this loop (Figure 4) and consequently their three-fold protrusions are separated (Figure 5) [9]. The intermediate phenotype seen in the TuV capsid is due to this loop being truncated by 6 amino acids compared to MVMp and adopting an alternate conformation (Figure 2, Figure 3a,c and Figure 4a). The 3-fold region of some protoparvoviruses plays a role in virus transduction and immunogenicity [48,49]. For CPV and FPV, cell infection requires the transferrin receptor (TfR) [50]. However, while TfR mostly binds to the 2/5-fold wall, an essential contact residue is located in VR1 (N93) [51]. This residue is not conserved in MVM, CuV, or TuV. The antigenic epitope for monoclonal antibody B7 against MVM includes amino acids located in VR3 and VR8 [43]. Additionally, a series of monoclonal antibodies bind at or near the 3-fold symmetry axis of CPV and FPV [48]. None of these antibodies are expected to bind to CuV or TuV because of sequence and structural variability.

Another highly immunogenic region of the *Protoparvovirus* capsid is the 2/5-fold wall composed of multiple VP surface loops. The outer wall facing the depression around the 5-fold channel contains VR4 and VR7 whereas the inner wall surrounding the 2-fold axis is formed by VR6, VR9, and partially VR3. Parts of VR9 also form the wall of the depression at the 2-fold axis (Figure 3a and Figure 4b). Of these loops VR6 and VR9 display the highest structural variability compared to MVM in both CuV and TuV, with Cα distance differences of up to 12 Å and local RMSDs between ~4 to 6 Å (Figure 3b, Table 2). Both CuV and TuV possess six or eight amino acid insertions in VR6 and four or three amino acid deletions in VR9 (Figure 3c). The shorter VR9 loop in CuV contributes to the previously described broader 2-fold depression (Figure 2). MVM and H1-PV capsids have been shown to bind to SIA for cell attachment, and the binding site has been mapped to the 2-fold depression [19,21]. Critical residues for SIA binding are located in VR6. In addition, other residues in the SIA binding pocket include those located in VR3, VR7, and VR9. CPV was also shown to bind to SIA [20]. For this virus, SIA binding is used for hemagglutination with the critical residue mapped to R377 located between VR6 and VR,7 and is surface exposed at the 2/5-fold wall.

In contrast to the other VRs, structural variability in VR5 is limited when comparing all available protoparvovirus structures (Figure 4a). While TuV’s VR5 topology is similar to MVMp, CuV displays some minor perturbations, up to a Cα-Cα difference of 2.8 Å (Figure 3b). However, VR5 is not surface exposed in CuV or any other *Protoparvovirus* (Figure 4). The structural variability seen here is probably compensating for the changes in VR3 and VR8 situated above this loop. Another region with minor structural variability is the AB-loop with Cα-Cα differences of up to 3.4 Å (Figure 3b). This difference, caused by a single deletion in CuV, is also shared with the BuVs but absent from all other known protoparvovirus structures. The AB-loop is located at the 2-fold axis in the interior of the capsid. X-ray crystal structures of parvoviruses with genome-containing capsids showed ordered DNA in this region [39,41,52,53]. Thus, these changes might control DNA binding in CuV and the BuVs.

### 3.4. BuV and CuV Are Closely Related

The structural similarities of CuV to the BuVs in VR8 resulting in the separated 3-fold protrusions (Figure 4 and Figure 5) and other similarities such as the AB-loop mentioned above, hint that these viruses are closely related. CuV shares the highest sequence identity with BuV2 in the ordered VP region at ~84%, followed by BuV1 and BuV3 (~65–67%) (Table 3). This is also the identity range between the other BuVs suggesting that perhaps CuV is a BuV. However, the NS protein is used for species determination and shows that these viruses do not belong to the same group. For all other protoparvoviruses, for which structures are available, the VP3 sequence identity with CuV is in the 30–33% range. With respect to structure, CuV aligns 94–96% with the BuVs and 70–79% with the other protoparvovirus structures (Table 3). TuV is equally different from the other protoparvoviruses compared, including the BuVs, with low sequence identity at 30–43% (Table 3). However, structural similarity is 74–82%. For MVMp, the highest amino acid sequence identity is with other rodent protoparvoviruses, LuIII and H1-PV (67–72%), which are also the most structurally similar (~97%). Interestingly, the non-human protoparvoviruses, with rodent, domesticated pet (CPV and FPV), and porcine hosts, share ~50% sequence identity and ~90% structural similarity.

### 3.5. TuV Binds to α2-3-Linked and Poly SIA while CuV Potentially Binds α2-6-Linked SIA

**Figure 6:**
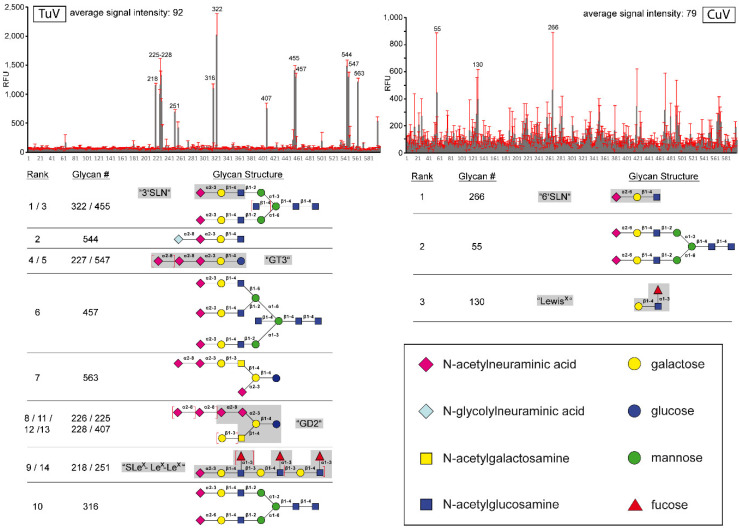
Glycan array of CuV and TuV. Relative fluorescence units (RFU) versus glycan number for TuV (top left) and CuV (top right) VLPs. 600 different glycan molecules (CFG glycan array v5.2) were screened. The gray bars represent the fluorescence detected for each glycan with the standard deviation shown in red. Below each histogram are symbol representation of the glycans with the highest signals. Trivial names of glycans are given where available. The definition of each symbol is provided on the bottom right hand side.

Sialic acid binding has been reported as the first attachment interaction for a number of protoparvoviruses, including MVM, H1-PV, CPV, FPV, and PPV [19,20,21,22]. For the human protoparvoviruses, there is no information regarding receptor usage. To this end, CuV and TuV capsids labeled with a fluorescent tag were analyzed on a glycan array with 600 different glycan molecules as previously reported for MVM and other parvoviruses [32,54]. The readouts from these arrays showed approximately the same average signal fluorescent intensity for both viruses with strong signals over background for TuV and weaker signals for CuV (~5-fold over background with large error bars) (Figure 6). In the TuV array the 17 top glycan binders all represent SIA containing carbohydrates (14 highest are shown in Figure 6). Among these SIA-containing glycans, three main types can be identified: (I) glycans with α2-3-linked SIA in a 3’SLN (*N*-acetylneuraminic acid-α2-3-galactose-β1-4-*N*-acetylglucosamine) context; (II) polysialic acids with α2-8-linkages; and (III) 3’SLNLNLN fucosylated at the *N*-acetylglucosamine (sLeXLeXLex). Glycans with α2-3-linked SIA are found on *N*-glycans, *O*-glycans, and glycolipids which are broadly expressed in mammals [55]. Glycans with α2-8 sialylation are found mainly in vertebrate brains and on a few glycoproteins in non-neuronal cells and on tumor cells [55]. The sLeX motif is found to be upregulated in tumor cells [56]. Binding to 3’SLN, α2-8 polysialated, and sLeX glycans were previously reported for MVMi while MVMp bound to 3’SLN and sLeX glycans [54]. Interestingly, TuV also bound to N-glycolylneuraminic acid containing glycans. These glycans do not exist in humans but are bound by FPV and CPV [20]. This, like the structure, further indicates that TuV is a “hybrid” virus between the primate and non-primate protoparvoviruses. The signals of the strongest binder in the CuV glycan array are weaker and more variable compared to TuV (Figure 6). Thus, only the glycans with signals at least 5-fold over background were analyzed. In contrast to TuV, the two highest signals showed glycans with α2-6-linked SIA in a 6’SLN (*N*-acetylneuraminic acid-α2-6-galactose-β1-4-*N*-acetyl- glucosamine) context (Figure 6). Glycans with α2-6-linked SIA are found on *N*-glycans, *O*-glycans, and glycolipids [55]. Other parvoviruses such as AAV1, AAV5, and AAV6 have been shown to bind α2-6-linked SIA [57,58]. Furthermore, the minimal LeX motif was weakly bound by CuV (Figure 6). Because of the low binding signals for CuV on the glycan array, these results are reported with caution. An expanded array with additional variants of the above glycans will be required to confirm these results and to find a glycan with higher binding affinity. However, despite the low signals, the glycan binding ability of CuV is clearly different from TuV.

### 3.6. Cell Binding Assays Confirm that SIA Serves as a Cellular Attachment Receptor for CuV and TuV

**Figure 7:**
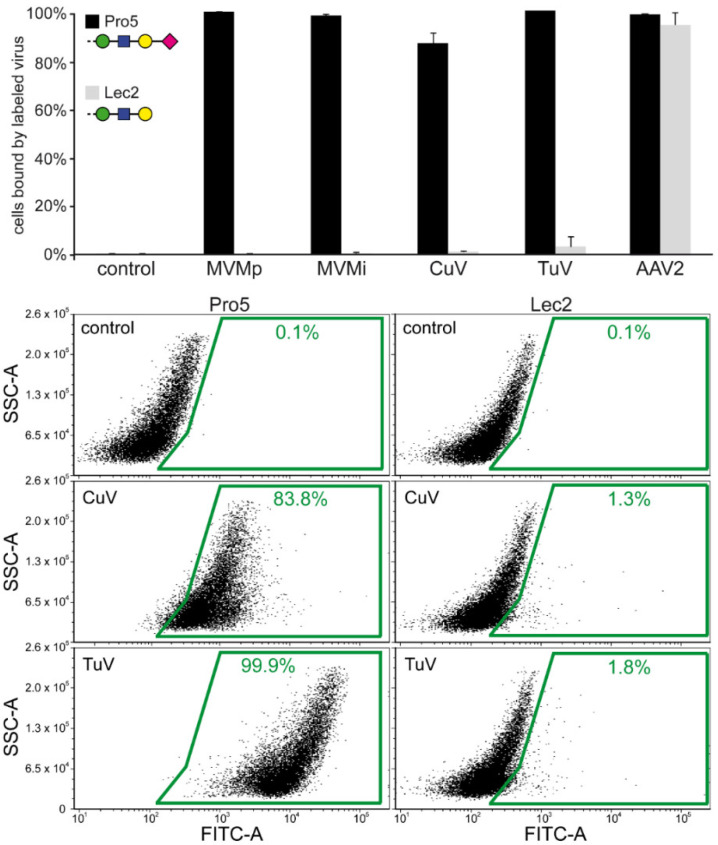
SIA is the attachment receptor for CuV and TuV. The percentage of cells with fluorescent signal are shown for the different viruses tested as a histogram. AAV2 was tested as a positive control for both cell lines. Shown below are example FACS histograms for control cells, and cells incubated with either fluorescently-labeled CuV or TuV capsids.

CuV and TuV binding to terminal SIA was confirmed with cell binding assays using differential glycan presenting CHO cell lines, Pro5 and Lec2 (Figure 7). The parental CHO-Pro5 cell line displays terminal SIA and the mutant Lec2 cell line displays terminal galactose, resulting from a mutation in a specific gene required for glycan biosynthesis [59]. AAV2 was tested as a positive control because it binds to heparan sulfate proteoglycan expressed in both cell lines (Figure 7). In contrast, MVM (tested alongside), CuV, and TuV bound efficiently to Pro5 cells but not to the SIA deficient Lec2 cell line (Figure 7). A similar assay, done previously for H1-PV, produced similar results [19]. SIA binding is common among the protoparvoviruses. This leads to the question whether or not they bind SIA in a similar location of the capsid. Currently, the only capsid-glycan complex structure available for a protoparvovirus is for MVMp with SIA [21]. In this study, the critical amino acids include I362 and K368 located in VR6 (Figure 3a,c). Other residues observed in close proximity to the SIA binding site were K241, M243, Y396, W398, D399, T401, F403, D553, Y558, and T578 located in VR3, VR7, and VR9. For H1-PV, capsid variants with I367S (aligns to MVMp I362) and H373R (aligns to K368) substitutions have loss of SIA binding [19]. This is surprising because MVMi possesses an arginine in position 368 [10] and binds to SIA (Figure 7) [54] which suggests that other residues play a role. The same mutation in MVMp (K368R) did not result in loss of SIA binding and added the ability to bind GT3 [54]. None of these amino acids is conserved in CuV or TuV except for I340 in TuV (aligns to MVMp I362). For CPV, a variant, CPV-R377K, lost SIA binding [60]. This arginine is structurally conserved in MVM (R375) and TuV (R361), located between VR6 and VR7 (Figure 3c) and surface exposed at the 2/5-fold wall. In contrast, CuV possesses a lysine in the same position (K377), which is similar to the CPV variant (R377K) unable to bind SIA. Thus, while TuV might bind SIA in a similar way to MVM or CPV, CuV’s SIA binding is likely different due to sequence and structural variability in the capsid’s surface loops. This lack of conservation also likely led to the weaker CuV binding in the glycan array.

## 4. Conclusions

This study reports the capsid structures for two of the most divergent viruses so far discovered in the *Protoparvovirus* genus. Based on the sequence and structure comparisons, TuV lies between the human protoparvoviruses, e.g., BuV and CuV, and the animal protoparvoviruses (H1-PV, LuIII, MVM, CPV, FPV, and PPV) (Figure 5). The most significant structural difference is the separated arrangement of the 3-fold protrusions of the human protoparvoviruses similar to other human parvoviruses (e.g., B19V, AAV2, and HBoV1), unlike the animal protoparvoviruses, which suggest a host-specific function for the 3-fold region. Despite the high structural variability, both CuV and TuV bound to SIA similar to the other protoparvoviruses. It remains unclear whether CuV and TuV utilize similar regions of the capsid for receptor binding especially considering the different specificity toward different SIA linkages and the weaker affinity of CuV. Thus, the reported capsid structures will serve as a 3D platform for functional characterization of CuV, TuV, and the protoparvoviruses in general. Furthermore, this study could inform efforts to develop antiviral strategies and vaccines for these pathogenic viruses.

## Figures and Tables

**Table 1:** Summary of data collection, image-processing, and refinement statistics for CuV and TuV.

**Cryo-EM Data and Refinement Parameter**	**CuV**	**TuV**
Total number of micrographs	738	580
Defocus range (µm)	0.90–3.84	0.83–4.27
Electron dose (e-/Å2)	75	75
Frames / micrograph	50	50
Pixel size (Å/pixel)	1.05	1.07
Capsids used for final map	15,296	33,191
Resolution of final map (Å)	2.87	2.88
**PHENIX model refinement statistics**		
Residue range	32–569	19–565
Map CC	0.874	0.866
RMSD bonds (Å)	0.01	0.01
RMSD angles (°)	0.86	1.01
All-atom clash score	8.10	7.66
**Ramachandran plot**		
Favored (%)	97.8	95.6
Allowed (%)	2.2	4.4
Outliers (%)	0	0
Rotamer outliers (%)	0.2	0.2
C-β deviations	0	0

**Table 2:** Overview Cα RMSDs for the superposed CuV, TuV, and MVMp VP structures.

RMSD [Å]	Overall	w/o VRs	AB- loop	VR1	VR2	VR3	VR4	VR5	VR6	VR7	VR8	HI- loop	VR9
**CuV vs. MVMp**	**3.0**	1.0	**2.5**	**4.1**	**4.1**	**6.6**	**2.2**	**2.0**	**5.9**	**3.1**	**5.2**	**2.0**	**6.3**
**TuV vs. MVMp**	**2.2**	0.8	1.1	1.6	**4.4**	**2.2**	**2.4**	0.9	**5.4**	**2.6**	**4.0**	**2.2**	**3.8**
**CuV vs. TuV**	**2.7**	1.0	**2.9**	**3.3**	**2.3**	**6.7**	1.9	1.4	**6.9**	1.4	**3.7**	**2.3**	**3.8**

**Table 3:** Protoparvovirus sequence (%, bottom left) and structural (%, top right) identity comparison.

	**BuV1**	**BuV2**	**BuV3**	**CuV**	**TuV**	**MVMp**	**LuIII**	**H-1PV**	**CPV**	**FPV**	**PPV**	Structural aligned amino acids: Cα distance < 2Å (in %)
**BuV1**	-	91.4	98.0	95.6	79.8	70.8	72.2	71.4	69.7	70.4	71.5	
**BuV2**	66.0	-	92.0	93.9	74.4	65.6	67.8	65.1	65.8	65.2	65.1	
**BuV3**	74.4	65.2	-	95.4	78.1	70.0	69.0	67.8	68.5	70.4	70.1	
**CuV**	67.0	83.6	64.7	-	79.3	72.1	71.8	71.4	71.5	70.8	69.9	
**TuV**	35.1	32.5	33.6	33.3	-	80.4	81.9	79.9	80.4	80.7	80.7	
**MVMp**	31.2	32.1	31.9	31.8	41.4	-	96.9	97.1	88.9	91.2	90.8	
**LuIII**	32.6	32.2	31.6	33.3	43.0	72.2	-	96.1	87.7	89.4	90.8	
**H-1PV**	32.9	31.2	32.9	30.4	41.6	67.4	68.4	-	88.7	91.6	90.9	
**CPV**	33.6	33.2	33.3	32.2	40.4	52.2	53.3	52.9	-	96.2	89.9	
**FPV**	32.3	32.3	32.6	31.8	40.4	52.4	53.1	52.5	98.0	-	90.0	
**PPV**	33.3	34.6	34.0	32.3	42.6	51.3	51.1	51.4	59.2	58.8	-	
	Amino acid sequence identity in ordered VP structure (in %)											

Abbreviations: BuV—bufavirus; CuV—Cutavirus; TuV—Tusavirus; MVMp—Minute of mice virus prototype strain; H-1PV—H-1 parvovirus; CPV—canine parvovirus; FPV—feline parvovirus; PPV—porcine parvovirus.
